# Grand Canonical Monte Carlo Simulations to Determine the Optimal Interlayer Distance of a Graphene Slit-Shaped Pore for Adsorption of Methane, Hydrogen and their Equimolar Mixture

**DOI:** 10.3390/nano11102534

**Published:** 2021-09-28

**Authors:** Jelle Vekeman, Daniel Bahamon, Inmaculada García Cuesta, Noelia Faginas-Lago, José Sánchez-Marín, Alfredo Sánchez de Merás, Lourdes F. Vega

**Affiliations:** 1 Instituto de Ciencia Molecular, Universitat de València, Catedràtic José Beltrán 2, 46980 Paterna, Spain; jose.sanchez@uv.es; 2 Alya Technology and Innovation SL, C/Republica 42, Sabadell, 08202 Barcelona, Spain; daniel.garcia@ku.ac.ae; 3 Research and Innovation Center on CO_2_ and H_2_ (RICH), Khalifa University of Science and Technology, Abu Dhabi P.O. Box 127788, United Arab Emirates; 4 Chemical Engineering Department, Khalifa University of Science and Technology, Abu Dhabi P.O. Box 127788, United Arab Emirates; 5 Departamento de Química Física, Universitat de València, Dr. Moliner 50, 46100 Burjassot, Spain; Inmaculada.Garcia-Cuesta@uv.es (I.G.C.); Alfredo.Sanchez@uv.es (A.S.d.M.); 6 Dipartimento di Chimica, Biologia e Biotecnologie, Università di Perugia, Consortium for Computational Molecular and Materials Sciences (CMS)2, Via Elce di Sotto 8, 06123 Perugia, Italy; noelia.faginaslago@unipg.it

**Keywords:** graphene, methane, hydrogen, grand canonical Monte Carlo, adsorption, slit-shaped pore

## Abstract

The adsorption—for separation, storage and transportation—of methane, hydrogen and their mixture is important for a sustainable energy consumption in present-day society. Graphene derivatives have proven to be very promising for such an application, yet for a good design a better understanding of the optimal pore size is needed. In this work, grand canonical Monte Carlo simulations, employing Improved Lennard–Jones potentials, are performed to determine the ideal interlayer distance for a slit-shaped graphene pore in a large pressure range. A detailed study of the adsorption behavior of methane, hydrogen and their equimolar mixture in different sizes of graphene pores is obtained through calculation of absolute and excess adsorption isotherms, isosteric heats and the selectivity. Moreover, a molecular picture is provided through z-density profiles at low and high pressure. It is found that an interlayer distance of about twice the van der Waals distance of the adsorbate is recommended to enhance the adsorbing ability. Furthermore, the graphene structures with slit-shaped pores were found to be very capable of adsorbing methane and separating methane from hydrogen in a mixture at reasonable working conditions (300 K and well below 15 atm).

## 1. Introduction

Methane and hydrogen gases have raised considerable attention because of the advantages they have over more traditional fossil fuels in the context of energy storage and delivery [1]. While the role of methane is probably limited to an—important—transition fuel that can easily be applied in existing technologies [2,3], hydrogen is becoming increasingly relevant in the context of sustainable energy [4]. Indeed, it is an excellent choice for long term energy storage in combination with renewable energy, while it also holds promise to decarbonize the transportation sector. However, it does come with problems related to safe and economical storage and transportation (which is currently done at very low temperatures and high pressures) [5,6]. Adsorption on porous materials may allow for storage and transportation of substantial amounts of H${}_{2}$ as well as CH${}_{4}$ at ambient conditions [7,8]. Aside from its role as transition fuel (while hydrogen-based technologies are being developed), methane exhaust from industrial and transport processes is a major challenge to society because of its large greenhouse effect. To reduce the emission of methane, materials are needed that can separate and capture the gas from exhaust mixtures [9]. Another process for which the separation of a methane/hydrogen mixture is of interest is the purification of H${}_{2}$ gas after production, while the methane/hydrogen mixture itself has shown promise to improve the energy delivery of pure methane or natural gas [10,11].

Clearly adsorbent materials are needed that can store (for transportation), capture or separate gases ranging from pure hydrogen to pure methane and mixtures of interest in between. Multiple materials have been suggested for this purpose such as metal organic frameworks [12,13], activated carbon [8,14], nanocarbon materials [15,16] and others [17]. Particularly interesting are graphene and derived materials, such as graphene oxide, [18] doped graphene [19] and defected graphene [20], relying on their van der Waals interaction with the gases leading to physisorption [21]. Indeed, because of its specific electronic properties and 2d-character, graphene has strong affinity for individual gas molecules [22]. A very interesting branch of graphene derivatives relies on careful tuning of the interlayer space between separate graphene sheets by introducing pillars made, for example, out of carbon nanotubes or fullerenes [23]. Very important for such efforts is to obtain a clear picture of the ideal distance between layers of graphene at different operating conditions, to accommodate as many molecules as possible. Therefore, in this work, different graphene -pores have been simulated using Grand Canonical Monte Carlo simulations to study the effect of different pore sizes on the adsorption of methane and hydrogen. More specifically, interlayer distances of 5 Å, 8 Å, 14 Å and 20 Å were considered.

As in all separation processes, the operating conditions are of utter importance for its applicability. In order to improve the current storage possibilities for hydrogen and to facilitate the processes described above, adsorption preferentially takes place at low pressures and around room temperature. This is essential because large pores can accommodate many molecules under high pressure, but will most likely be less selective [24]. Smaller pores with tuned interaction energies may render the need for high pressures less important, relying more on the intrinsic selectivity of a pore for the adsorbate [25]. To take this notion into account, the different considered pores were simulated in a pressure range between 0 atm and 70 atm.

In many molecular simulation studies, such systems are modeled using the Lennard–Jones potential to represent the dispersion interactions, despite the known limitations-at both long and short distance range-of this potential [26,27]. In some cases, an electrostatic energy term is added to further improve the picture via allocation of partial charges on the molecules [28,29]. In this work, the Lennard–Jones potential, was replaced by the so-called Improved Lennard–Jones (ILJ) potential, introduced by Pirani et al. [30,31]. It contains an extra parameter in the exponent of the repulsive term to add the needed flexibility to better describe the intermediate distance range of the interaction. The parameters for such potentials are usually obtained by fitting to experimental data and are assumed-which is not always justified-to be very transferable from one system to another [32,33]. On the contrary, the parameters used in this work were specifically developed for the adsorption of gas molecules on graphene by fitting to dimer interaction energies at density functional theory (DFT) level [34,35,36,37].

The ILJ potential has been extensively tested in molecular dynamics (MD) simulations with success in the past years, but has never been used in a Grand Canonical Monte Carlo (GCMC) setting to the best of our knowledge [36,38,39]. While MD is a very useful tool, the information obtainable from GCMC is complementary as the phase space is more adequately explored, especially for gas adsorption [40,41,42]. Furthermore, GCMC allows for simulation without a fixed number of particles in the system which is particularly interesting for the description of experimental setups like gas adsorption in a pore [43,44]. As such, this work will be trialling the use of the ILJ potential in GCMC calculations to further exploit the better performance of the ILJ potential in specific systems of industrial interest, such as the adsorption and separation of methane and hydrogen using graphene with pores of different sizes at a large pressure range.

The paper is oulined as follows: In Section 2, the computational details of the work are discussed together with the ILJ potential and used models. Results are presented in Section 3 and some conclusions are presented in Section 4.

## 2. Computational Details

GCMC simulations were performed by means of the LAMMPS package [45,46] with an in-house implementation of the ILJ potential. The pores were represented by two parallel sheets of graphene in their usual honeycomb-like structure [47,48], consisting of 1660 carbon atoms each with a C-C bond distance of 1.42 Å. Pillars to maintain the interlayer distance were omitted and graphene layers were maintained rigid-atomic positions fixed at the initial positions-during the whole simulation. Periodic boundary conditions were applied in the x- and y-directions with interlayer distances of 5 Å, 8 Å, 14 Å and 20 Å along the z-axis. Because of this set-up, an infinite pore is simulated and no hydrogens were introduced to cap off graphene edges, while in the z-direction, the box size was taken such that the box walls coincided with the graphene planes. All simulations were performed at a temperature of 300 K and in a pressure range between 0 atm and 70 atm, typical operating conditions for such adsorption processes. As is usual in GCMC simulations, the volume, temperature and chemical potential-controlled via the pressure-were taken as independent thermodynamic variables. Three kinds of moves were considered to construct the Markov chain: Particle deletion, particle insertion and particle displacement, all with equal probability, whereby a particle is understood to be either methane or molecular hydrogen. A total of 5 × 10${}^{5}$ configurations were simulated to reach a converged uptake of the gas molecules as an ensemble average, while a cutoff distance of 14 Å was used and convergence was further monitored via properties like temperature and energy. Adsorption isotherms were then obtained by plotting the evolution of the uptake at different pressures.

For the adsorption isotherms, both the absolute and excess amounts of molecules were calculated whereby the latter was obtained by subtracting the amount that would be present in a bulk gas with the same volume, from the absolute amount adsorbed

(1) $$n_{i}^{e}=n_{i}^{a}-\rho V_{\mathrm{pore}}y_{i},$$

where $n_{i}^{e}$ and $n_{i}^{a}$ are the excess and absolute amount adsorbed, respectively, of compound *i*. The absolute amount adsorbed of a given simulation is the number of molecules that are present in the simulation box after convergence. $V_{\mathrm{pore}}$ is the pore volume, which equals the volume of the simulation box, $y_{i}$ is the mole fraction of compound *i* and ρ is the gas density at the specified pressure and temperature as calculated by the Soave-Redlich-Kwong equation of state [49].

It has been experimentally verified that methane adsorption obeys the Toth equation [50]

(2) $$n_{i}^{a}=q_{i}^{m}\frac{k_{i}P}{{\left[1+{\left(k_{i}P\right)}^{t}\right]}^{\frac{1}{t}}},$$

where *P* is the pressure, $q_{i}^{m}$ is the adsorption capacity at saturation, $k_{i}$ is the Langmuir equilibrium constant of compound *i* and *t* is a fitting parameter. The value of t as extrapolated from the data in ref. [50] is 0.91 at 300 K, justifying the use of a simpler Langmuir model. Actually, the inherent deficiencies of this model (e.g., the surface must be a perfectly flat plane with no corrugations, there is a mono-layer coverage only and there are no interactions between adsorbate molecules and adjacent adsorption sites) can be essentially disregarded in the present systems as the adsorbent is composed of pristine graphene sheets, the adsorption coverage is not too large and the interaction between adsorbate molecules is very weak [34,37]. In fact, this model has already been used in this context [21,51,52,53]. Therefore, the obtained absolute adsorption isotherms from GCMC were fitted to the Langmuir equation

(3) $$n_{i}^{a}=q_{i}^{m}\frac{k_{i}P}{1+k_{i}P}.$$

This choice is further validated by the quality of the Langmuir fittings with $R^{2}$ values of 0.985 and higher, as discussed below.

Density profiles of the pure components and mixtures were calculated as the amount of molecules falling within spatial bins in the z-direction of the simulation box as an average over time, while the selectivity was calculated using

(4) $$S_{a,b}=\frac{{\left(x_{a}/x_{b}\right)}_{\mathrm{pore}}}{{\left(x_{a}/x_{b}\right)}_{\mathrm{bulk}}},$$

where *x* is the mole fraction and *a* and *b* represent different molecules (i.e., CH${}_{4}$ and H${}_{2}$).

Isosteric heats of adsorption were calculated using the fluctuation method [54]

(5) $$Q_{m}=\frac{\langle U\rangle \langle N\rangle -\langle UN\rangle }{\langle N^{2}\rangle -\langle N\rangle \langle N\rangle }+k_{b}T,$$

where the angle brackets denote ensemble averages, *U* is the configuration energy of the system, *N* the number of particles, $k_{b}$ is the Boltzmann constant and T the temperature.

The force field used to model the gas-gas and the graphene-gas interactions assumes that the intermolecular interactions are pairwise additive and both the gas molecules and graphene sheets are rigid (all optimized at the B3LYP/631G** DFT level of theory). The only considered contribution to the intermolecular interaction is a van der Waals term, namely the ILJ potential [30,31]

(6) $$V\left(R\right)=\epsilon \left[\frac{m}{n\left(R\right)-m}{\left(\frac{r_{0}}{R}\right)}^{n\left(R\right)}-\frac{n\left(R\right)}{n\left(R\right)-m}{\left(\frac{r_{0}}{R}\right)}^{m}\right],$$

where

(7) $$n\left(R\right)=\beta +4\left(\frac{R}{r_{0}}\right).$$

Here, ϵ and $r_{0}$ represent the potential well and its location, while β is a dimensionless parameter that provides extra flexibility for fitting to the interaction energy curve. The β value is loosely related to the hardness of the interacting systems and is usually confined to an interval between 7 and 9 (although this may be lowered for systems with relatively strong electrostatic interactions), while *m* is 6 in all cases [55]. Note that the improvement of the ILJ potential over the LJ potential has been demonstrated elsewhere [31,34,55].

The interaction parameters for the methane and hydrogen molecules and their interactions with graphene were taken from previous publications and are given in Table 1 [34,37]. For completeness, the principle of the fitting procedure is shortly described, while more details can be found in the original papers. A large amount of randomly generated dimer interaction energies were obtained at DFT-level and different suitable potentials were fitted to this data. The quality of these potentials was checked by benchmarking against CCSD(T) and DFT (using the B97-D level of theory) calculations on a set of symmetric dimers which were not present in the fitting data. After, the performance of the potentials was further tested in MD simulations by calculating diffusion coefficients, which is directly comparable to experiment [34]. While different models were fitted in these previous works whereby the ILJ potential was complemented with a Coulombic sum (testing different charge schemes), the cheapest considered model, a united-atom model without partial charges, was found to show good performance for methane and hydrogen [34,37]. Therefore, this latter, computationally cheaper model (with a single interaction center and no atomic charges) was chosen for the investigations performed in the current work. It should be stressed that because of the fitting to DFT-level interaction energies, the electrostatic and other possible interaction types are implicitly taken into account in the potential. Indeed, this procedure has proven successful in several MD studies [36,38,39]. The interaction parameters for the methane/hydrogen interaction, were developed specifically for this work following the same procedure described before and thus, no combination rules were used.

## 3. Results and Discussion

**Figure 1:**
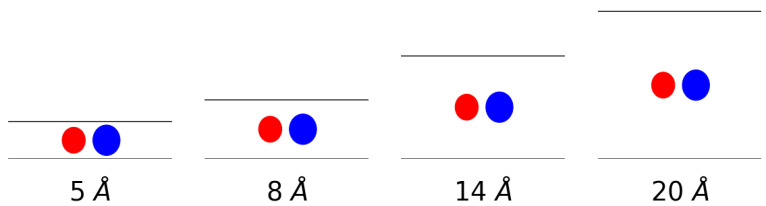
A schematic representation of the gas molecules within the considered pores. The spheres represent the van der Waals radii of methane (blue) and hydrogen (red) and the black lines represent the graphene sheets.

The gas uptake of molecular hydrogen and methane and an equimolar mixture of both in a slit-shaped pore was simulated. The pores were composed of two graphene sheets whereby the interlayer distance was varied to simulate different pore sizes. More specifically, interlayer distances of 5 Å, 8 Å, 14 Å and 20 Å were considered. This means that very narrow pores, about the size of the adsorbates, were simulated on one end and pores with a size of about five times the van der Waals radius of the adsorbates on the other. In Figure 1, a schematic representation is given of the van der Waals radii-taken as the equilibrium distance of the force field-of both methane and hydrogen in comparison to the size of the pores indicated by horizontal lines.

### 3.1. Adsorption Behavior

**Figure 2:**
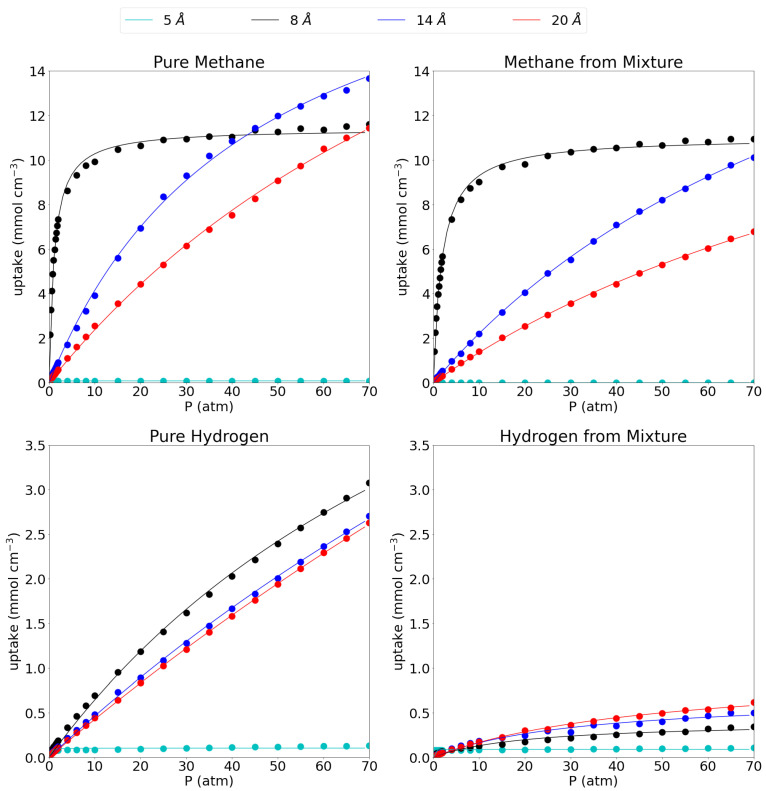
Absolute adsorption isotherms for pure methane (**top left**), methane from the equimolar mixture (**top right**), pure hydrogen (**bottom left**) and hydrogen from the equimolar mixture (**bottom right**) at 300 K. The dots represent calculated values, while the full lines show the fitted Langmuir isotherm. Please notice the different scales on the y-axes.

In Figure 2, the absolute adsorption isotherms for the respective pore sizes are shown for methane and hydrogen, both in their respective pure phases as well as in their equimolar mixture. In the main text, only volumetric adsorption isotherms are shown, while the gravimetric isotherms can be found in SI as the behavior is rather similar for all systems. In this work, results obtained at 1 atm, representing low pressure conditions, are compared to results obtained at 70 atm as a representation of extreme operating conditions. In this reasoning, the absolute uptakes of the pores at 1 atm and 70 atm are given in Table 2.

When considering the pure methane gas first, it is seen that the behavior of the adsorption isotherm is drastically different for the considered pore sizes. The smallest pore, the 5 Å pore, shows nearly no adsorption of methane gas as is seen by the near-zero absolute adsorption isotherm which remains flat along the entire pressure range (0.08 mmol cm${}^{-3}$ at both low and high pressure). The 8 Å pore shows a very fast saturation with increasing pressure. Indeed, at 1 atm it has already reached 5.50 mmol cm${}^{-3}$, about half of its maximum capacity (11.61 mmol cm${}^{-3}$ at 70 atm) and at 15 atm it has already reached close to its maximum capacity meaning that raising the pressure further does not accommodate many more molecules. For the larger pores of 14 Å and 20 Å, the convergence is slower with rising pressure and no plateau is reached within the investigated pressure range. Indeed, at 1 atm they have only reached uptakes of 0.50 mmol cm${}^{-3}$ and 0.34 mmol cm${}^{-3}$, respectively, while at 70 atm, they adsorb 13.66 mmol cm${}^{-3}$ and 11.44 mmol cm${}^{-3}$, respectively. The absolute adsorption isotherms of the 14 Å and the 20 Å pore cross the 8 Å pore isotherm at 40 atm and 70 atm, respectively, adsorbing a larger amount of molecules per unit of volume above those pressures. Such behavior was also reported before by Chen [56] et al. From this, it seems that below 40 atm, the 8 Å pore is the most efficient one, while above 40 atm, the 14 Å becomes more efficient and it remains as such within the remainder of the investigated pressure range.

Lin et al. [21] have reported adsorption isotherms of methane on graphene slit-like pores with interlayer distances of 20 Å and beyond. They found the same trend of lowering of the molar density with increasing pore size and also found Langmuir behavior, indicating a single adsorption layer on every graphene sheet, as is also reflected in our adsorption isotherms. The late convergence of the larger pores is also reflected in their adsorption isotherm which starts to converge at around 250 atm. Importantly they have not included pores smaller than 20 Å despite the increased adsorption capacity of methane with decreasing pore width. They also did not study hydrogen adsorption nor the methane/hydrogen mixture. The absolute adsorption capacity of the pores can also be compared to a series of MOFs as reported by Zhang et al. [57] who reported the highest adsorption for the NJU-Bai structure with a total uptake of 6.48 mmol cm${}^{-3}$ at 64.15 atm. This means that the most efficient pore in this work (i.e., 8 Å) considerably outperforms the reported MOFs and already reaches similar uptakes at pressures as low as 2 atm. Gravimetrically, the difference is even more striking as the considered pores outperform a series of MOFs as reported by Korman et al. [58] by an order of magnitude. Indeed, the highest value they found was 0.14 mmol g${}^{-1}$ at 1.2 atm compared to 2.96 mmol g${}^{-1}$ (see Supplementary Materials).

The picture is quite similar for methane in the equimolar CH${}_{4}$/H${}_{2}$ mixture. Indeed the 5 Å pore still shows close to no adsorption of methane (0.002 mmol cm${}^{-3}$ at 1 atm and 0.0004 mmol cm${}^{-3}$ at 70 atm). The 8 Å pore shows very similar behavior as for the adsorption of the pure methane gas, although the adsorption uptake does lower slightly to 3.98 mmol cm${}^{-3}$ at 1 atm and 10.93 mmol cm${}^{-3}$ at 70 atm. Moreover, the large pores do behave slightly different upon adsorption of the mixture as the methane adsorption isotherm on the 14 Å pore is no longer crossing the 8 Å isotherm at around 40 atm, but is set to do so just outside the investigated pressure range. It shows uptakes of 0.29 mmol cm${}^{-3}$ and 10.11 mmol cm${}^{-3}$ at 1 atm and 70 atm, respectively, again slightly lower than for the pure methane. Likewise, the 20 Å pore sees its adsorption of methane molecules shifted to higher pressures with the uptake at 1 atm dropping to 0.19 mmol cm${}^{-3}$ at 1 atm and 6.78 mmol cm${}^{-3}$ at 70 atm.

For the hydrogen gas (please note the different scale on the y-axis compared to the adsorption isotherms for methane), it is seen that, as for methane, the smallest pore adsorbs only a very limited amount of pure hydrogen, 0.08 mmol cm${}^{-3}$ at 1 atm and 0.13 mmol cm${}^{-3}$ at 70 atm. For the larger pores, the 8 Å pore proves the most effective in absolute numbers with adsorption uptakes of 0.12 mmol cm${}^{-3}$ (1 atm) and 3.08 mmol cm${}^{-3}$ (70 atm). Crucially, the uptake is always much lower than for methane, especially at low pressures. The 14 Å and 20 Å pore are very similar in their behavior with uptakes of 0.07 mmol cm${}^{-3}$ and 0.06 mmol cm${}^{-3}$, respectively, at 1 atm and 2.70 mmol cm${}^{-3}$ and 2.63 mmol cm${}^{-3}$, respectively at 70 atm.

Looking at the hydrogen uptake from the methane/hydrogen mixture, it is seen that there is a strong influence from methane on the adsorption behavior of hydrogen for the three largest pores. Indeed, the presence of methane seriously hinders the adsorption of hydrogen, lowering the molecular uptake substantially to 0.05 mmol cm${}^{-3}$ for the 8 Å pore, 0.04 mmol cm${}^{-3}$ for the 14 Å pore and 0.03 mmol cm${}^{-3}$ for the 20 Å pore at 1 atm and 0.35 mmol cm${}^{-3}$, 0.50 mmol cm${}^{-3}$ and 0.62 mmol cm${}^{-3}$ at 70 atm, respectively. Interestingly, the hydrogen uptake behavior as a function of the pore size is now reversed compared to the pure hydrogen gas as the largest pore adsorbs the highest amount of hydrogen per unit of volume. This relates to the lost selectivity of larger pores at high pressures, whereby all available molecules are pushed into the available volume. The 5 Å pore adsorbs 0.08 mmol cm${}^{-3}$ and 0.11 mmol cm${}^{-3}$ at 1 atm and 70 atm, respectively. These numbers are very similar as for the pure hydrogen gas given the very low adsorption of methane from the mixture. Taken together with the results for the methane gas from the mixture, this means that a graphene-based adsorbent, with an interlayer distance of 8 Å is the most efficient one for methane capture and separation from the equimolar mixture over the entire pressure range.

Langmuir models were fitted to the adsorption isotherms for the three larger pores (i.e., 8 Å, 14 Å and 20 Å) for which the parameters are given in Table 3. The smallest studied pore of 5 Å was left out since the very low uptake numbers do not allow for a reasonable fitting of a Langmuir isotherm. Indeed, with the exception of the 5 Åpores, all Langmuir fittings showed R${}^{2}$ values of at least 0.985. It is seen that, for methane, the adsorption capacity rises with increasing interlayer distance, while the Langmuir equilibrium constant, *k*, decreases. Since a larger *k* constant indicates a strong interaction with the solid material, this suggests that better adsorption will be possible in smaller pores. The same trends are observed for hydrogen, but the equilibrium constants are substantially smaller as could be inferred from the adsorption isotherms themselves. For the mixture, it is seen that the presence of the other gas, clearly influences the fitting parameters of the isotherm. For the methane gas, $q^{m}$ behaves quite similar for the 8 Å and the 14 Å pores, while it is smaller in the mixture than in the pure gas for 20 Å pores. In general, the equilibrium constants are lower in the equimolar mixture, indicating a less favorable adsorption for methane in the mixture. For the hydrogen molecule, the $q^{m}$ parameters are one order of magnitude higher in the pure gas than in the mixture, while the equilibrium constants are one order of magnitude lower.

**Figure 3:**
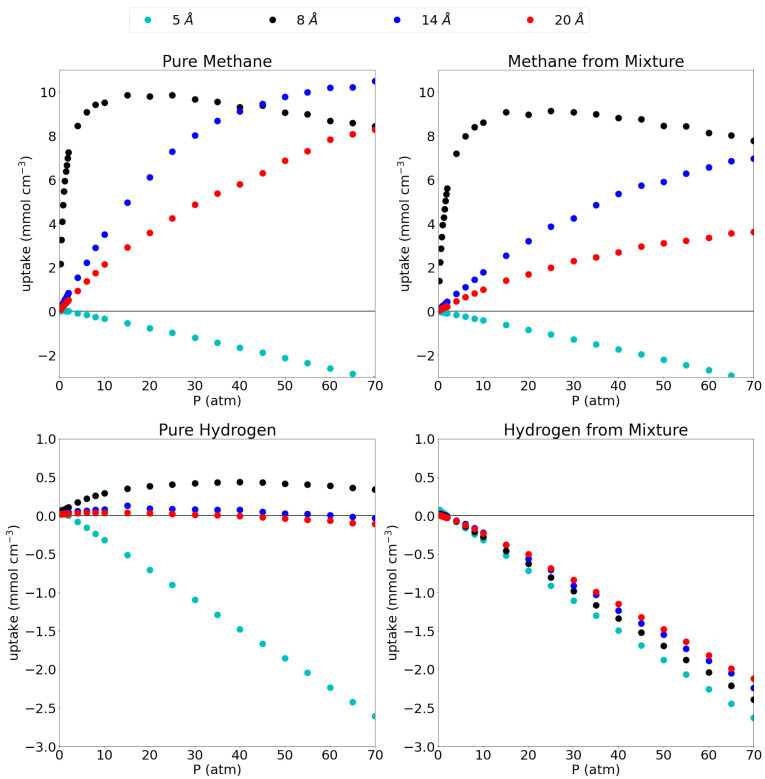
Excess adsorption isotherms for pure methane (**top left**), methane from the equimolar mixture (**top right**), pure hydrogen (**bottom left**) and hydrogen from the equimolar mixture (**bottom right**) at 300 K. Please notice the different scales on the y-axes.

A comparison between the uptake in the pore and the possible uptake of bulk gas in an empty container, is given as excess adsorption isotherms in Figure 3 and the explicit values at 1 atm and 70 atm can be found in Table 4. As explained before, the plots give the uptake in the pore that is higher (in excess) than the uptake in an empty container (i.e., bulk gas). This thus allows a good assessment of the pores’ efficiency to capture and store the studied gases.

For pure methane, it is seen that the 5 Å pore, which showed close to no adsorption, has an excess amount of molecules adsorbed of 0.04 mmol cm${}^{-3}$ and −3.08 mmol cm${}^{-3}$ at 1 atm and 70 atm, respectively. This indicates that the presence of the graphene sheets is actually prohibiting methane molecules to enter the simulation box, rather than adsorbing them. Looking at the equilibrium distance of the C${}_{\mathrm{graph}}$-CH${}_{4}$ interaction, the methane molecule prefers to maintain an ideal distance of 3.938 Å to either graphene sheet which is not possible in a pore of only 5 Å. Moreover, the closeness of the methane to the graphene sheets forces the molecules out of the pore through repulsive interactions. From the remaining pores, it is clear that all three of them have a higher molar density than the bulk phase as is evidenced by the strongly positive excess adsorption isotherms at high pressures with uptakes of 8.44 mmol cm${}^{-3}$, 10.50 mmol cm${}^{-3}$ and 8.27 mmol cm${}^{-3}$ at 70 atm for the 8 Å, 14 Å and 20 Å pores, respectively. At low pressures, the 8 Å pore is seen to be very efficient with an excess uptake of 5.46 mmol cm${}^{-3}$ at 1 atm, whereas the 14 Å and 20 Å pore adsorb only 0.46 mmol cm${}^{-3}$ and 0.29 mmol cm${}^{-3}$, respectively.

As already indicated by the absolute adsorption isotherms, the adsorption efficiency slightly lowers when going from pure methane to methane from the mixture. Indeed, these numbers drop slightly to −0.04 mmol cm${}^{-3}$, 3.93 mmol cm${}^{-3}$, 0.25 mmol cm${}^{-3}$ and 0.15 mmol cm${}^{-3}$ in increasing pore size order, at 1 atm and to −3.17 mmol cm${}^{-3}$, 7.77 mmol cm${}^{-3}$, 6.95 mmol cm${}^{-3}$ and 3.61 mmol cm${}^{-3}$ at 70 atm. Interestingly, the excess adsorption isotherm for the 8 Å pore for both pure methane and methane from the mixture, show a negative gradient above 15 atm (the pressure whereby the pore is saturated), indicating that the density rises faster with increasing pressure in an empty container than in the pore. At this point, the gas molecules would prefer to stay in the bulk volume over going inside the pore as they will lose entropy by being confined at this pressure. Similar trends were found for a 0.8/0.2 CH${}_{4}$/H${}_{2}$ mixture in carbon cilindrical cavities, where indeed, larger pores showed lower adsorption and only the smallest pores maxima in their excess adsorption isotherms. [15] Differently to our results, however, they reported substantial methane adsorption in a pore of only 4.6 Å diameter; note that these were not slit-shaped pores though and the geometry of the pore plays a key role at these small pore sizes.

For pure hydrogen in the 5 Å pore, the excess adsorption is mostly strongly negative (0.04 mmol cm${}^{-3}$ at 1 atm, but −2.63 mmol cm${}^{-3}$ at 70 atm) meaning that, although the pore does adsorb hydrogen, there is a lot less hydrogen present in the pore than there would be in the same volume of bulk hydrogen. The 8 Å pore shows a slight advantage compared to the bulk molar density, although this advantage is only present at low pressures and slowly dwindles with increasing pressure as can be seen by the maximum that is present in the excess adsorption isotherm, uptakes of 0.08 mmol cm${}^{-3}$ and 0.34 mmol cm${}^{-3}$ are reported for pure hydrogen at 1 atm and 70 atm and −2.39 mmol cm${}^{-3}$ for hydrogen from the mixture at the same pressures. For the larger pores, it seems like the graphene barely influences the amount of hydrogen gas in the simulation box, as the large size of the pore compared to the hydrogen molecules (and the relatively weak interaction with graphene), leaves the hydrogen molecule as in bulk. For the 14 Å pore, uptakes of 0.03 mmol cm${}^{-3}$ and −0.005 mmol cm${}^{-3}$ are found at 1 atm for the pure hydrogen and hydrogen from the mixture, while −0.03 mmol cm${}^{-3}$ and −2.24 mmol cm${}^{-3}$ are found at 70 atm. The values for the 20 Å pore are almost equal with an excess adsorption of 0.02 mmol cm${}^{-3}$ and −0.11 mmol cm${}^{-3}$ for the pure hydrogen at 1 atm and 70 atm, respectively, and −0.01 mmol cm${}^{-3}$ and −2.12 mmol cm${}^{-3}$ at the same pressures for hydrogen from the mixture. The above results for the absolute adsorption isotherms are confirmed in the sense that adsorption of hydrogen from the gas mixture is strongly disfavored, leading to strongly negative excess adsorption isotherms. The smallest pore (i.e., 5 Å) shows an excess adsorption of 0.04 at 1 atm and −2.63 at 70 atm, while the 8 Å pore shows an adsorption of 0.01 at 1 atm and −2.39 at 70 atm. The two largest pores (14 Å and 20 Å) have an excess adsorption of −0.005 mmol cm${}^{-3}$ and −0.01 mmol cm${}^{-3}$ at 1 atm, respectively and −2.24 mmol cm${}^{-3}$ and −2.12 mmol cm${}^{-3}$ at 70 atm. A similar qualititative result was reported by [15] for hydrogen adsorption from a 0.2/0.8 CH${}_{4}$/H${}_{2}$ mixture on carbon cilindrical cavities: Lowering excess adsorption curves with increasing pore size, which reach a maximum at relatively low pressures.

**Figure 4:**
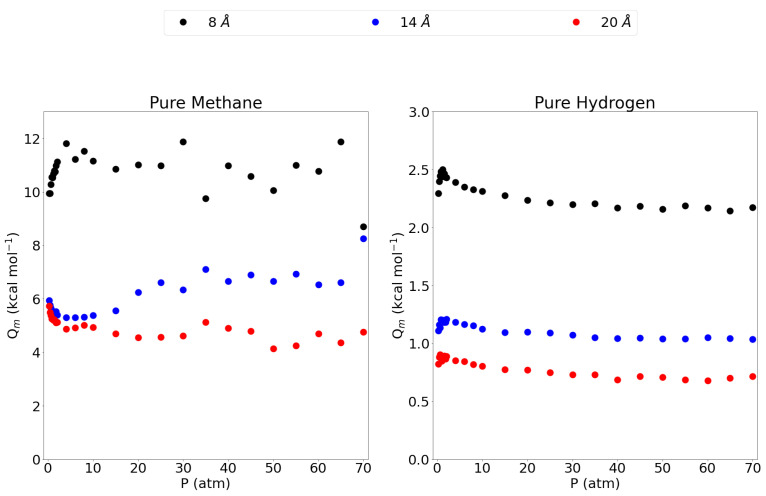
The isosteric heat of adsorption for methane and molecular hydrogen within the investigated pores and at the entire pressure range considered and at 300 K. Please notice the different scales on the y-axes.

Figure 4 shows the isosteric heats of adsorption, as a function of pressure, for the pure methane gas and the pure hydrogen gas in the 8 Å, 14 Å and 20 Å pores. The 5 Å pore is left out since the isosteric heat is very close to 0 as almost no methane gas is adsorbed in this pore. The 8 Å pore shows a high isosteric heat compared to the 14 Å and 20 Å pores, indicating a stronger adsorption of the methane molecule in this pore as observed before. Interestingly, the isosteric heat is about twice the size of the larger pores which may be explained by the fact that in the 8 Å pore, the methane molecules are in ideal position to adsorb to both graphene sheets at the same time leading to an interaction that is twice as strong. For the larger pores, the molecules adsorb to only one sheet and feel a lower influence from the opposing graphene sheet. This is further confirmed by the observation that the 14 Å shows a slightly higher isosteric heat than the 20 Å pore, the closeness of the opposing graphene sheet, seems to substantially influence the isosteric heat. For hydrogen, the three larger pores all show a maximum at lower pressures, lowering into a plateau at higher pressures. Again, the 8 Å pore has by far the highest isosteric heat, followed by the 14 Å and then the 20 Å pore. While the adsorption isotherm showed little difference between these three pores, the difference is clear in the isosteric heat of adsorption. Again, these results are significantly higher than values reported in the literature for zeolites, MOFs and activated carbons. Indeed, a comparison between some adsorbents is made in the work by Abdulsalam et al. [59] and only some types of multi-walled carbon nanotubes are reported to have isosteric heats ($Q_{m}$ = 9.91 kcal mol${}^{-1}$) close to the value reported for the 8 Å pore in this work. The remaining adsorbents all have isosteric heats lower than 7 kcal mol${}^{-1}$.

The results that were so far presented allow drawing some conclusions on the adsorption behavior of pure methane, pure hydrogen and their equimolar mixture on the investigated pores. First of all, the slit-shaped pores show a clear preference for the adsorption of methane over hydrogen. Indeed, at the chosen temperature, much more pure methane is adsorbed into the pores than pure hydrogen with the exception of the smallest pore, which is too small to accommodate methane molecules. Secondly, there is a correlation between the closeness of the opposing graphene sheet and the affinity of the pore to adsorb gas molecules due to overlapping of either graphene sheet with the gas molecule. Indeed, it is suggested that a slit-shaped pore with the size of twice the van der Waals interaction of the adsorbate is most ideal as it maximizes the interaction with both pore walls. This was also noted by Rzepka et al. and Kuchta et al. [60,61] for the adsorption of molecular hydrogen in a 7 Å pore. While such a pore was not considered in this work, it can be inferred that a 7 Å pore would outperform the 8 Å pore in terms of hydrogen adsorption. However, the difference is not expected to be large enough to make the considered slit-pores very attractive for hydrogen adsorption. Thirdly, large pressures are needed in order for the larger pores to outperform the smaller ones as their adsorption isotherm saturates a lot slower with rising pressure. These conclusions hold for both methane and hydrogen, even though the latter has less affinity for the investigated pores. Indeed, for hydrogen it was already found in the past that pristine graphene pores are not able to adsorb large amounts of molecular hydrogen at room temperature [62]. However, in a context of gas separation as is under investigation here, this can be beneficial if the difference in adsorption capacities of both molecules is sufficiently large as is shown below. Finally, it is important to bare in mind that the temperature plays a key role in adsorption, although its effect was outside the scope of this study as the focus was specifically on room temperature conditions for ease of application. Indeed, 300 K is a very high temperature for hydrogen compared to its critical point, while it is a relatively low temperature for methane. As such, different performance results may be obtained at other temperatures as has been established in the past by Bénard et al. [63]. Indeed, they found that the adsorption of hydrogen on pristine graphene pores can reach considerable levels at low temperatures.

**Figure 5:**
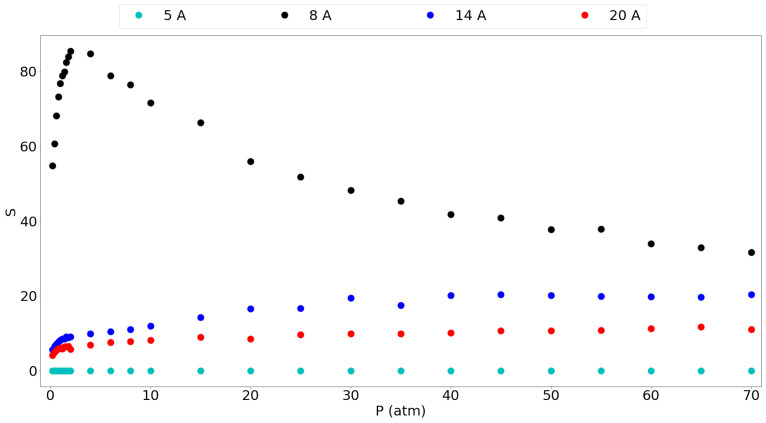
Selectivity for the different pores of methane over hydrogen for an equimolar mixture in the entire considered pressure range.

Figure 5 gives the selectivity of methane over hydrogen for the different pores considered. It is seen that the 8 Å gives by far the highest selectivity and that it shows a peak at around 2 atm, to be 85.3. Furthermore, the selectivity at 1 atm is still very high at 76.68 thus suggesting again that a graphene slit pore of 8 Å is very suitable for methane separation from hydrogen. The larger pores also show lower selectivities without a maximum, where it is clear that larger pores show a smaller preference for methane over hydrogen. This confirms the idea that graphene-based materials with tuned larger pores are less selective and allow easier entrance of the hydrogen molecules in this case. The 5 Å pore is a special case which favors the adsorption of hydrogen based on molecular size, thus giving a selectivity very close to 0, although the small amount of H${}_{2}$ molecules entering keeps it from being very useful as mentioned before.

Kumar et al. [11] have reported equimolar methane/hydrogen selectivities for different graphite slit-pore widths at 298 K. They found the same trend of decreasing selectivity with increasing pore size, although they found that the selectivity decreased with rising pressure for a given pore. In our results, the selectivity rises slightly with the pressure and then seems to stabilize. Similar to our results, they found an extremely high selectivity, around 250, for the methane molecule in a 7.3 Å pore which is comparable to our 8 Å pore having a very high selectivity. This selectivity drops very fast when increasing the pore size already below 20 for a 12 Å pore. Furthermore, they reported a selectivity of around 10 for the 15 Å pore and around 8 for the 19 Å pore. These results are in the same range as our 14 Å and 20 Å pore results. The differences in the values in their work and this work may be explained by the use of a generic force field with Lorentz-Berthelot mixing rules, while this work used a tailor-made force field.

### 3.2. Molecular Picture

**Figure 6:**
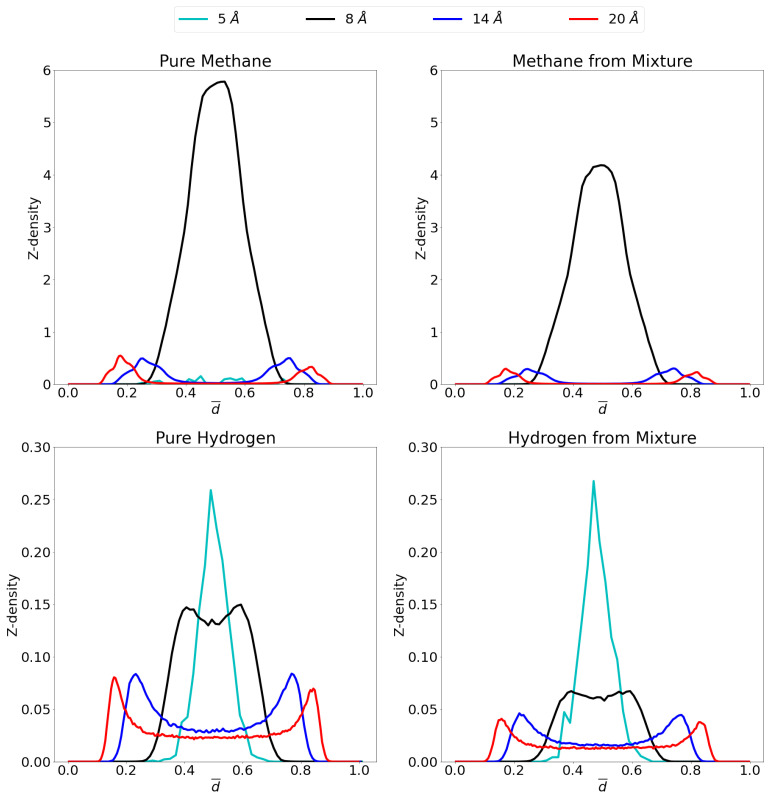
Z-density profiles at 1 atm for pure methane (**top left**), methane in the mixture (**top right**), pure molecular hydrogen (**bottom left**) and molecular hydrogen from the mixture (**bottom right**) in the different considered pores at 300 K. Reduced distance units are used so that the graphene sheets of the pores are located at 0 and 1 on the x-axis. Please notice the different scales on the y-axes.

**Figure 7:**
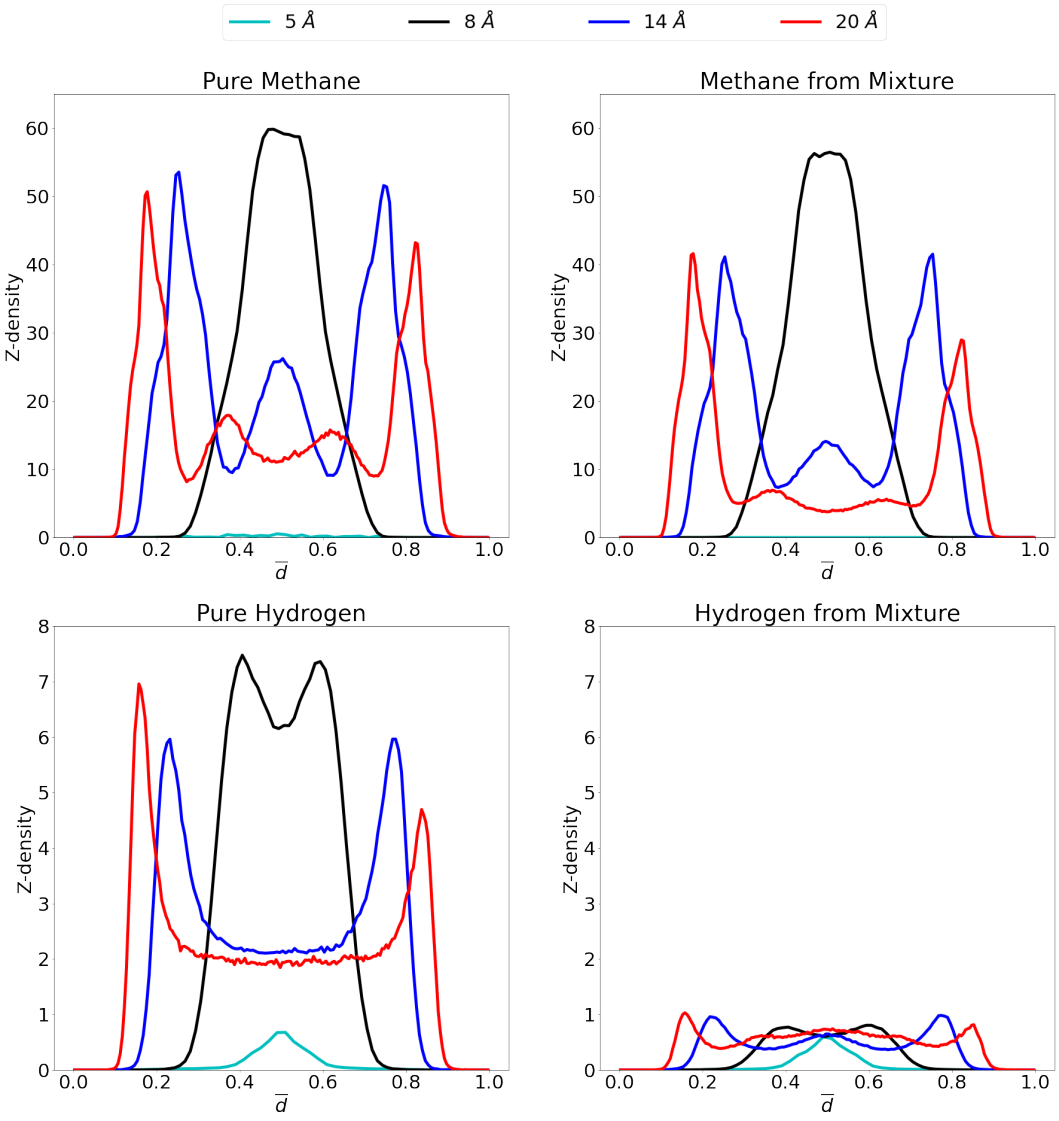
Z-density profiles at 70 atm for pure methane (**top left**), methane in the mixture (**top right**), pure molecular hydrogen (**bottom left**) and molecular hydrogen from the mixture (**bottom right**) in the different considered pores at 300 K. Reduced distance units are used so that the graphene sheets of the pores are located at 0 and 1 on the x-axis. Please notice the different scales on the y-axes.

In order to gain insight on the molecular distribution of the gas molecules within the pores, z-density profiles were calculated as shown in Figure 6 at 1 atm and Figure 7 at 70 atm. To ease comparison, reduced distance units were used meaning that the two graphene sheets are located at 0 and 1 on the x-axis. More specifically, the distance, *r*, is divided by the respective interlayer distances to obtain the reduced distance, $\bar{d}$. Relevant snapshots with the host molecules configurations for these simulations are given in Supplementary Materials at both 1 atm and 70 atm.

For pure methane at a pressure of 1 atm, it is seen that close to no molecules are adsorbed in the 5 Å pore, while a single, strong adsorption layer is present in the middle of the 8 Å pore. For the larger pores, two different adsorption layers are found, one on either graphene sheet. It is seen that the adsorption layers for the 14 Å pore are positioned slightly further from the graphene sheets than in the 20 Å pore. This is presumably caused by the opposing graphene sheet in the 14 Å pore being closer than in the 20 Å pore and pulling the adsorption layer slightly further from the ideal position to the closest graphene sheet. The simulated molecular distribution is very similar in nature as in the simulations reported by Yu et al. [64] for a 10 Å and 20 Å pore as well as the center of mass probability distributions for methane adsorbed in graphene reported by Collins et al. [65]. For the mixture, all pores behave very similar as for the pure gas. For the 5 Å pore, there is still barely any methane adsorption, while the 8 Å, 14 Å and 20 Å pores show the same qualitative behavior as for the pure methane, but at slightly lower quantities.

For the adsorption of pure hydrogen in the 14 Å and the 20 Å pores, two adsorption layers are seen close to the graphene sheets, but there is a substantial gas phase in the middle, where gas molecules are free to behave as bulk hydrogen. This bulk phase is not present in the 8 Å pore because of a lack of space, the small size of the hydrogen molecule does allow for the presence of two overlapping adsorption layers. The 5 Å pore, conversely, shows one single adsorption layer in the middle of the pore. In the mixture the quantitative picture is very different for the three largest pores as the presence of methane strongly influences the hydrogen adsorption. However, qualitatively, the situation is quite similar with still 2 overlapping adsorption layers in the 8 Å pore, a single adsorption layer in the 5 Å pore and two separate layers with a bulk gas phase in the middle, for the largest pores.

It is interesting to note that the competition in the larger pores is stronger and more hydrogen is able to enter the pore compared to the 8 Å pore. This is caused by space left in the middle of the pore by methane. Indeed, it is clearly seen that in the middle of the pore, the space is occupied by hydrogen molecules, more than methane as has been observed previously [15]. This is due to the preferential adsorption of methane into graphene versus hydrogen, as already inferred from Figure 5 (selectivity curves), while hydrogen preferentially takes up the middle region of the pore.

At 70 atm there is a very large increase in the amount of methane molecules that can enter the respective pore. Indeed, the single layer of methane in the 8 Å pore shows an increase by a ten-fold in the amount of molecules that adsorb. Likewise for the 14 Å and 20 Å pores, the largest adsorption layers increase considerably, while a third adsorption layer is found in the former and even a fourth in the latter. Indeed, there is now a large proportion of methane molecules present in the middle of the pore, contrary to the situation at 1 atm, which forms second adsorption layers (when seen from the viewpoint of the graphene sheet) instead of forming bulk gas as was the case for hydrogen. In the 5 Å pore, there is still no considerable adsorption found, even at this high pressure, due to steric effects as already mentioned.

It was shown in the previous sections that the higher the pressure, the lower the advantage of the graphene pore becomes compared to an empty container at the same pressure. Again, this behavior can be understood by looking at the z-density profile and considering the equilibrium distance for the graphene-methane interaction, the pore of 8 Å allows methane molecules to enter the pore and sit in a position where the centre of mass is located more or less at the ideal distance from both graphene sheet feeling optimal attraction from both. There is, however, only a small region where this is possible and once this layer is saturated, the amount of molecules that further enter the system lowers. Newly entering molecules will now be forced to be placed closer to the graphene sheets, costing more energy. For the larger pores of 14 Å and 20 Å, the saturation of the pore requires larger pressures. There are now two ideal positions for the methane molecules, at either graphene sheet. As these ideal positions gets filled up, there is still room left in the middle of the pore to take up less ideal positions.

For hydrogen, it is interesting that, whereas at 1 atm, the 5 Å pore showed the highest adsorption peak, at 70 atm this is replaced by the 8 Å pore, where an overlapping double layer is found. In general, as expected, much larger quantities of molecules are adsorbed under these high pressures. It is also notable that no third or fourth adsorption layer is observed in the larger pore, but rather a large bulk gas is present in the middle of the pore reinforcing the results that, at these conditions of temperature and pressure, hydrogen is not attracted by graphene.

Interestingly, the effect of the high pressure is rather limited on the behavior of the methane gas within the mixture. Whereas it could be expected that large pressures make any molecule adsorb, rendering the pore less selective, this effect is quite limited. Indeed, the z-density profile for methane in the mixture is rather similar to the profile of the pure methane gas. Conversely, the hydrogen is significantly prohibited to adsorb in the pore in the presence of methane, leaving only small amounts of adsorption. The exception being of course the 5 Å pore, where methane molecules do not enter and thus not hinder the hydrogen adsorption.

## 4. Conclusions

In this work, the adsorption of methane, hydrogen and their equimolar mixture in graphene structires with slit-shaped pores of different sizes was investigated at a large pressure range and a temperature of 300 K using GCMC simulations and a tailor-made ILJ force field. Specific attention was dedicated to the role of the pore size, by means of varying the interlayer distance (between 5 Å and 20 Å) between the graphene pore walls. Absolute and excess adsorption isotherms, isosteric heats, selectivities and z-density profiles were calculated.

The obtained adsorption isotherms showed that the 8 Å, 14 Å and 20 Å pores are capable of adsorbing a large amount of methane, substantially outperforming an empty container of the same size. Thereby, the 8 Å pore was the most efficient one at pressures below 40 atm and the 14 Å was most efficient at higher pressures leading to an isosteric heat of about twice the value compared to that obtained for larger pores. For pure hydrogen systems at these conditions, only the 8 Å pore showed some benefit compared to an empty container, while the 14 Å and 20 Å pores barely influenced the presence of hydrogen in the simulation box. The smallest considered pore of 5 Å pore proved too be to narrow to adsorb substantial amounts of methane and/or hydrogen. For the mixture, it was shown that methane is strongly favored over hydrogen since almost no hydrogen was adsorbed in all considered pores and the methane adsorption was only hindered to a small extent by the present hydrogen. This is very good, for instance, to remove CH${}_{4}$ as impurities from a hydrogen stream.

The isosteric heats of adsorption showed a decreasing trend with increasing pore size due to the diminishing interaction of the adsorbate with the opposing graphene sheets. Indeed, in the 8 Å pore, the methane molecules are capable of positioning themselves at the ideal distance of either graphene sheet. For larger pores, the adsorbed molecule is forced to ’choose’ between either graphene sheet and the interaction with the other sheet diminshes with increasing interlayer distance. This is also reflected in the z-density profiles where it is seen that the adsorption layers on a given graphene sheet are located closer to that graphene sheet when the interlayer distance increases and the attraction of the opposing sheet dwindles. In accordance with the adsorption isotherms, the isosteric heat for hydrogen is about 4 to 5 times lower than for methane.

Given the clear preference of the pore walls for methane over hydrogen, a very high selectivity was found for the 8 Å, 14 Å and 20 Å pores. Especially the 8 Å pore showed a selectivity of over 80 at relatively low pressures, but also the larger pores show selectivities above 10 over the entire pressure range. Overall, the use of fine-tuned graphene pores seems very promising for the separation of methane/hydrogen mixtures at reasonable operating conditions. Given the use of more advanced pore materials at optimized interlayer distances, such as graphyne, gyroid materials, pillared materials or square pores, the adsorption and separation capacities may be further increased.

## Figures and Tables

**Table 1:** The interaction parameters used in this work.

System	ϵ (kcal mol${}^{-1}$)	r${}_{0}$ (Å)	β	Ref.
CH${}_{4}$-CH${}_{4}$	0.421	4.169	8.216	[34]
CH${}_{4}$-H${}_{2}$	0.180	3.816	5.618	This work
H${}_{2}$-H${}_{2}$	0.091	3.552	5.618	[37]
C${}_{\mathrm{graph}}$-CH${}_{4}$	0.210	3.938	8.185	[34]
C${}_{\mathrm{graph}}$-H${}_{2}$	0.088	3.527	6.813	[37]

**Table 2:** The absolute uptakes in the bulk in mmol cm${}^{-3}$ for the pure methane, pure hydrogen and both in their equimolar mixture at pressures of 1 atm and 70 atm (between brackets), both at 300 K.

Pore Size (Å)	Pure CH${}_{4}$	Pure H${}_{2}$	CH${}_{4}$ Mixt.	H${}_{2}$ Mixt.
5	0.08 (0.08)	0.08 (0.13)	0.00 (0.00)	0.08 (0.11)
8	5.50 (11.61)	0.12 (3.08)	3.98 (10.93)	0.05 (0.35)
14	0.50 (13.66)	0.07 (2.70)	0.29 (10.11)	0.04 (0.50)
20	0.34 (11.44)	0.06 (2.63)	0.19 (6.78)	0.03 (0.62)

**Table 3:** Parameters for a fitted Langmuir isotherm against the calculated values from Figure 2. $q^{m}$ in mmol cm${}^{-3}$ and *k* in atm${}^{-1}$.

Pore	Pure CH${}_{4}$		Pure H${}_{2}$		CH${}_{4}$ Mixt.		H${}_{2}$ Mixt.	
	$q^{m}$	k	$q^{m}$	k	$q^{m}$	k	$q^{m}$	k
8 Å	11.405	0.909	7.833	0.009	11.023	0.536	0.405	0.048
14Å	22.470	0.023	13.094	0.004	24.420	0.010	0.716	0.029
20 Å	31.178	0.008	18.170	0.002	19.898	0.007	0.989	0.020

**Table 4:** The excess uptakes in mmol cm${}^{-3}$ for the pure methane, pure hydrogen and both in their equimolar mixture at pressures of 1 atm and 70 atm (between brackets), both at 300 K.

Pore Size (Å)	Pure CH${}_{4}$	Pure H${}_{2}$	CH${}_{4}$ Mixt.	H${}_{2}$ Mixt.
5	0.04 (−3.08)	0.04 (−2.60)	−0.04(−3.17)	0.04 (−2.63)
8	5.46 (8.44)	0.08 (0.34)	3.93 (7.77)	0.01 (−2.39)
14	0.46 (10.50)	0.03 (−0.03)	0.25 (6.95)	−0.00 (−2.24)
20	0.29 (8.27)	0.02 (−0.11)	0.15 (3.61)	−0.01 (−2.12)

## Supplementary Materials

The following are available online at https://www.mdpi.com/article/10.3390/nano11102534/s1, Figure S1: Gravimetric adsorption isotherms at 1 atm for pure methane, pure hydrogen and their equimolar mixture. Figure S2: Gravimetric adsorption isotherms at 70 atm for pure methane, pure hydrogen and their equimolar mixture. Figures S3–S23: Snapshots at 1 atm for pure methane, pure hydrogen and their mixture. Figure S4: Snapshots at 70 atm for pure methane, pure hydrogen and their mixture.
