# Supplementary material for: Grand Canonical Monte Carlo Simulations to Determine the Optimal Interlayer Distance of a Graphene Slit-Shaped Pore for Adsorption of Methane, Hydrogen and their Equimolar Mixture
Source: Nanomaterials (Basel). 2021 Sep 28;11(10):2534. doi: 10.3390/nano11102534 (PMC8536989; doi:10.3390/nano11102534)
Supplement: Supplementary file 1 [file nanomaterials-11-02534-s001.zip › nanomaterials-1380889-supplementary.pdf]

Supplementary Materials: Grand Canonical Monte Carlo Simulations to Determine the Optimal Interlayer Distance of a Graphene Slit-Shaped Pore for Adsorption of Methane, Hydrogen and their Equimolar Mixture

Jelle Vekeman [1,2,*,†], Daniel Bahamon [2,3,4], Inmaculada García Cuesta [5], Noelia Faginas-Lago [6], José Sánchez-Marín [1], Alfredo Sánchez de Merás [5] and Lourdes F. Vega [2,3,4,*]

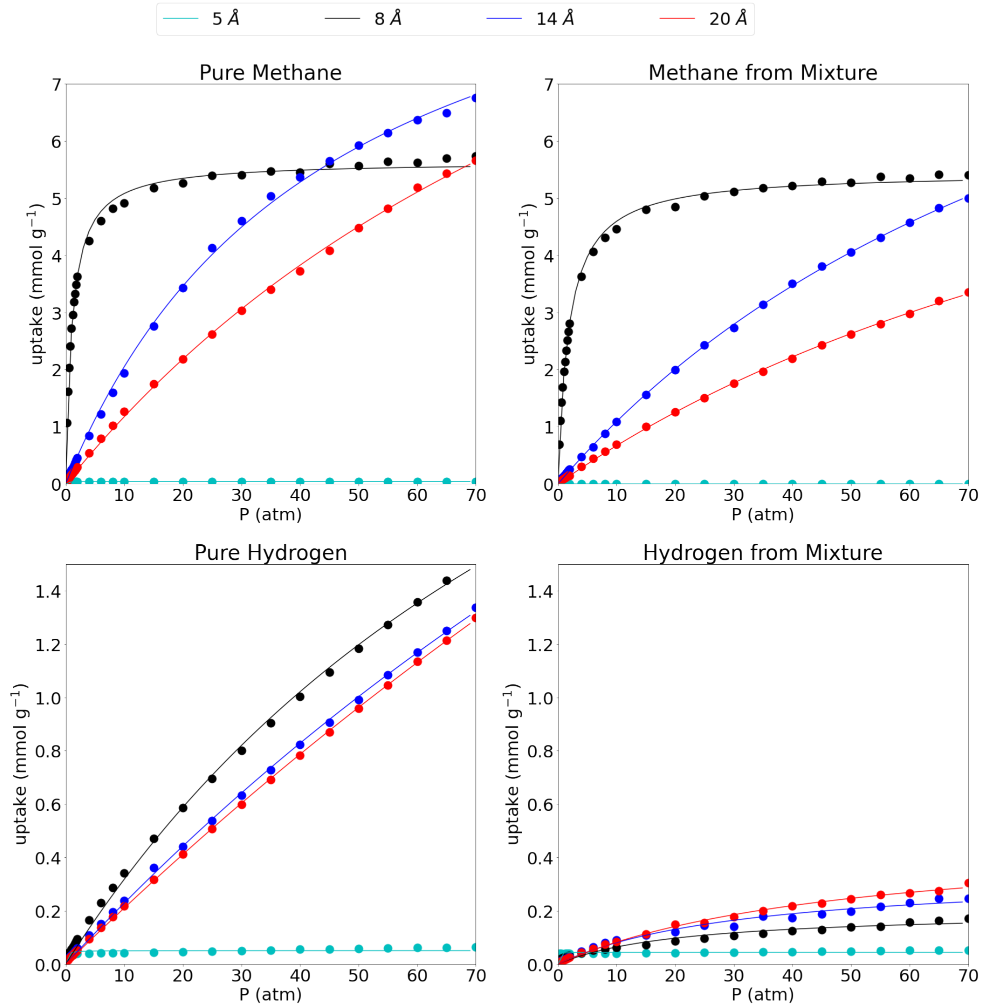

Figure S1. Absolute, gravimetric adsorption isotherms for pure methane (top left), methane from the equimolar mixture (top right), pure hydrogen (bottom left) and hydrogen from the equimolar mixture (bottom right) at 300 K. The dots represent calculated values, while the full lines show the fitted Langmuir isotherm. Please notice the different scales on the y-axes.

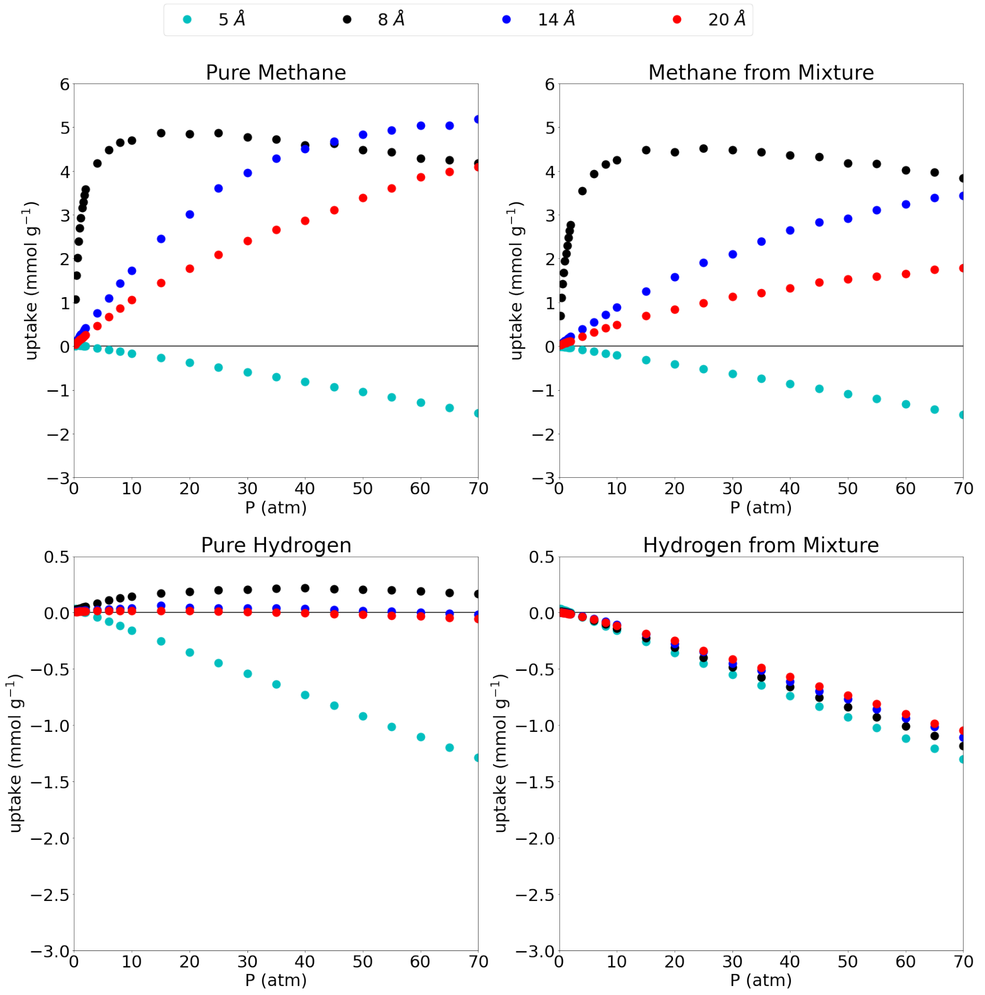

Figure S2. Excess, gravimetric adsorption isotherms for pure methane (top left), methane from the equimolar mixture (top right), pure hydrogen (bottom left) and hydrogen from the equimolar mixture (bottom right) at 300 K. Please notice the different scales on the y-axes.

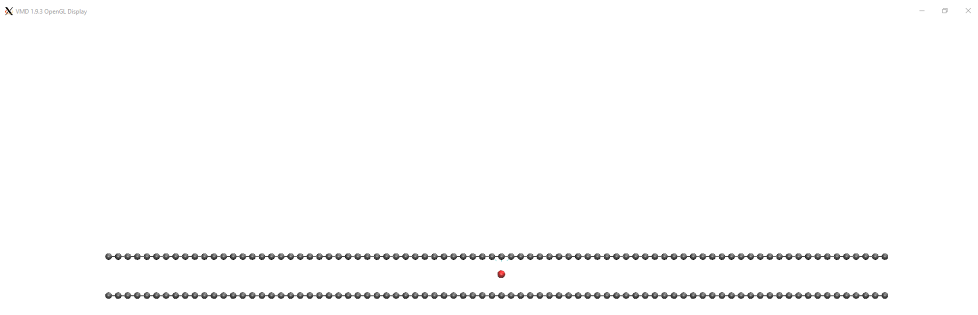

Figure S3. Molecular snapshot for CH_4 adsorption on the graphene slit-shaped pore with an interlayer distance of 5 Å at a temperature of 300 K and a pressure of 1 atm. For clarity, the center of mass of the methane molecules is pictured only (red).

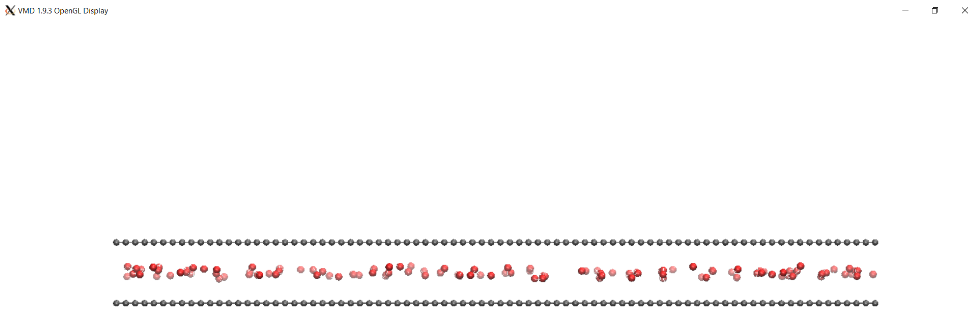

Figure S4. Molecular snapshot for CH_4 adsorption on the graphene slit-shaped pore with an interlayer distance of 8 Å at a temperature of 300 K and a pressure of 1 atm. For clarity, the center of mass of the methane molecules is pictured only (red).

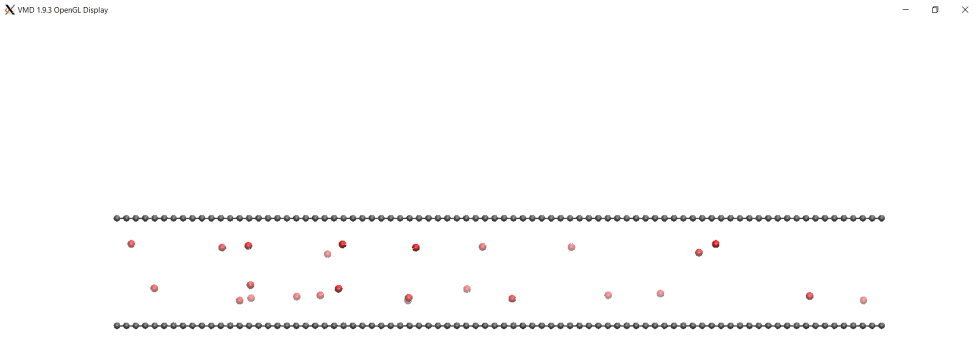

Figure S5. Molecular snapshot for CH_4 adsorption on the graphene slit-shaped pore with an interlayer distance of 14 Å at a temperature of 300 K and a pressure of 1 atm. For clarity, the center of mass of the methane molecules is pictured only (red).

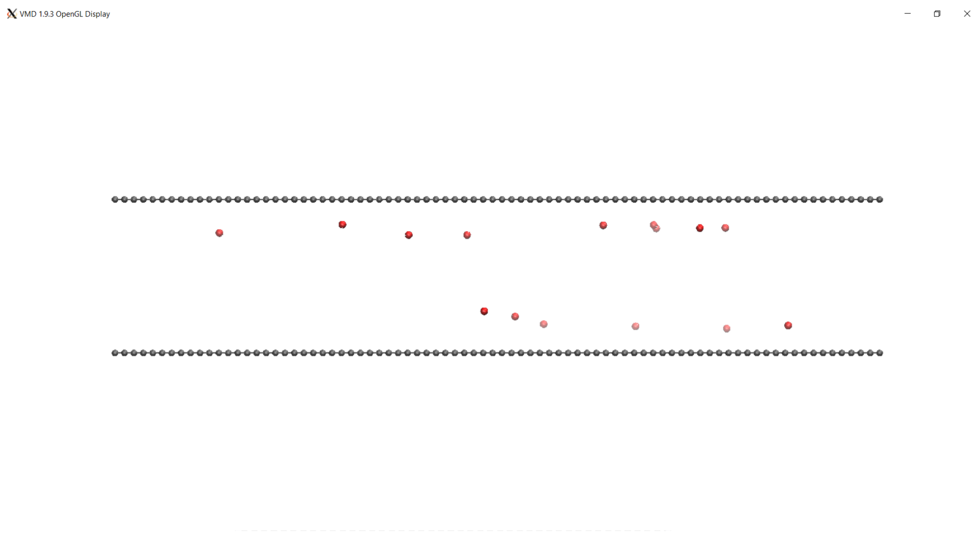

Figure S6. Molecular snapshot for CH_4 adsorption on the graphene slit-shaped pore with an interlayer distance of 20 Å at a temperature of 300 K and a pressure of 1 atm. For clarity, the center of mass of the methane molecules is pictured only (red).

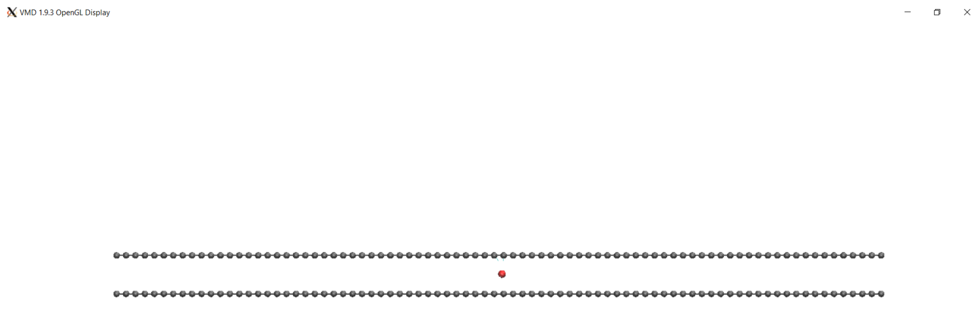

Figure S7. Molecular snapshot for CH_4 adsorption on the graphene slit-shaped pore with an interlayer distance of 5 Å at a temperature of 300 K and a pressure of 70 atm. For clarity, the center of mass of the methane molecules is pictured only (red).

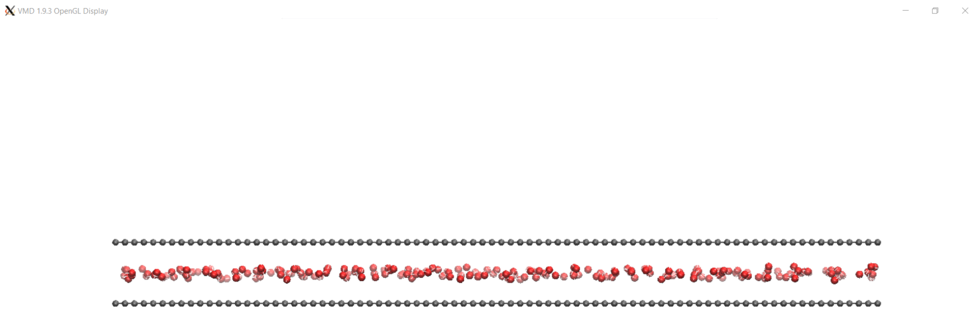

Figure S8. Molecular snapshot for CH_4 adsorption on the graphene slit-shaped pore with an interlayer distance of 8 Å at a temperature of 300 K and a pressure of 70 atm. For clarity, the center of mass of the methane molecules is pictured only (red).

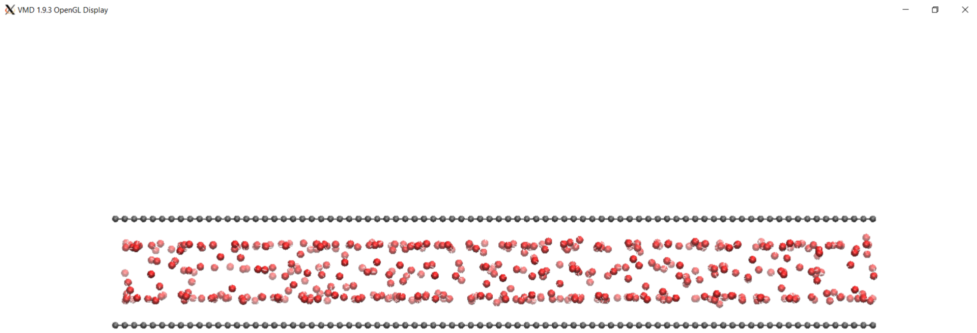

Figure S9. Molecular snapshot for CH_4 adsorption on the graphene slit-shaped pore with an interlayer distance of 14 Å at a temperature of 300 K and a pressure of 70 atm. For clarity, the center of mass of the methane molecules is pictured only (red).

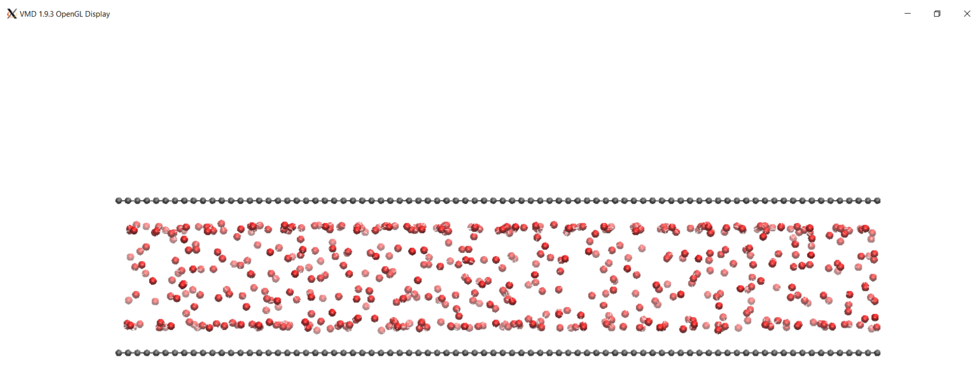

Figure S10. Molecular snapshot for CH_4 adsorption on the graphene slit-shaped pore with an interlayer distance of 20 Å at a temperature of 300 K and a pressure of 70 atm. For clarity, the center of mass of the methane molecules is pictured only (red).

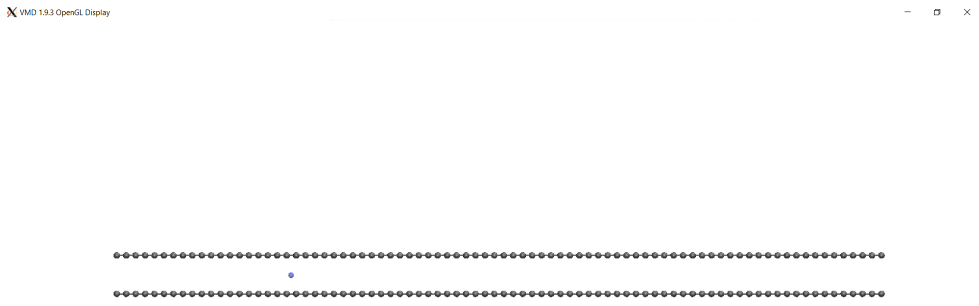

Figure S11. Molecular snapshot for H_2 adsorption on the graphene slit-shaped pore with an interlayer distance of 5 Å at a temperature of 300 K and a pressure of 1 atm. For clarity, the center of mass of the hydrogen molecules is pictured only (blue).

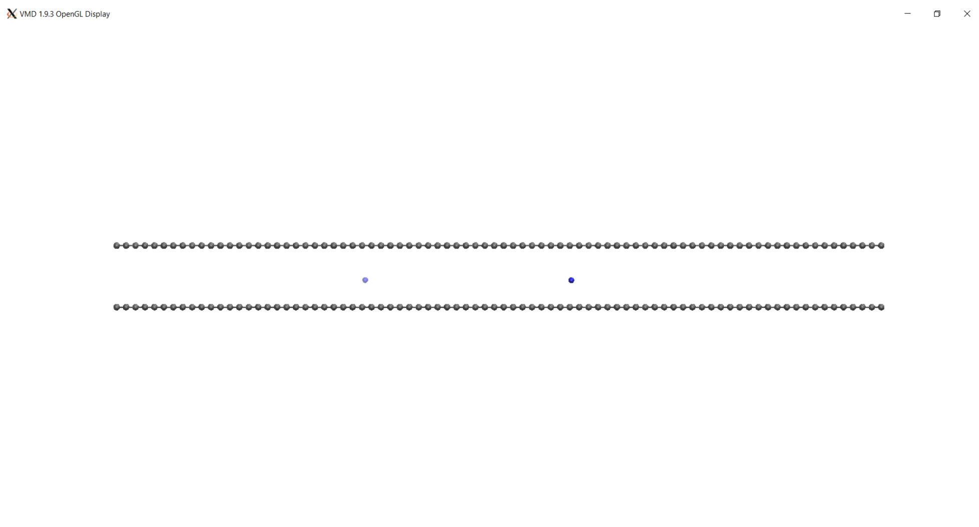

Figure S12. Molecular snapshot for H_2 adsorption on the graphene slit-shaped pore with an interlayer distance of 8 Å at a temperature of 300 K and a pressure of 1 atm. For clarity, the center of mass of the hydrogen molecules is pictured only (blue).

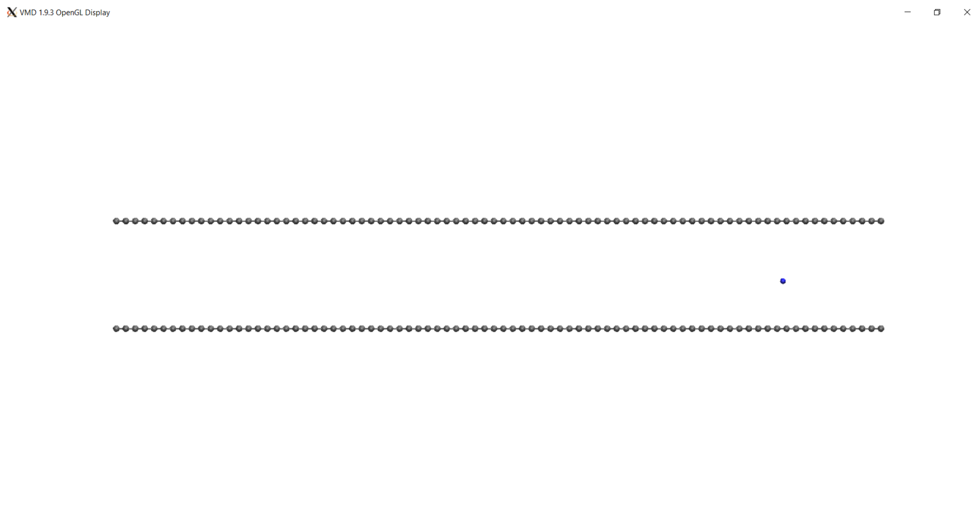

Figure S13. Molecular snapshot for H_2 adsorption on the graphene slit-shaped pore with an interlayer distance of 14 Å at a temperature of 300 K and a pressure of 1 atm. For clarity, the center of mass of the hydrogen molecules is pictured only (blue).

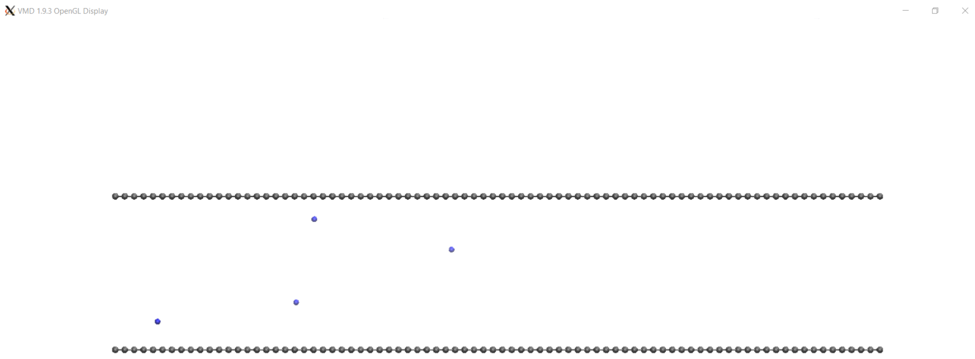

Figure S14. Molecular snapshot for H_2 adsorption on the graphene slit-shaped pore with an interlayer distance of 20 Å at a temperature of 300 K and a pressure of 1 atm. For clarity, the center of mass of the hydrogen molecules is pictured only (blue).

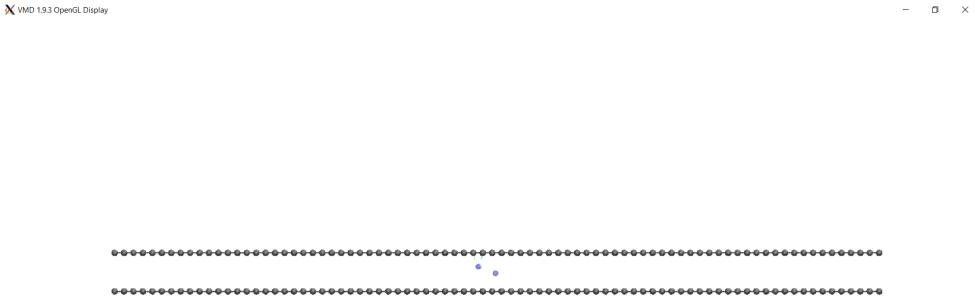

Figure S15. Molecular snapshot for H_2 adsorption on the graphene slit-shaped pore with an interlayer distance of 5 Å at a temperature of 300 K and a pressure of 70 atm. For clarity, the center of mass of the hydrogen molecules is pictured only (blue).

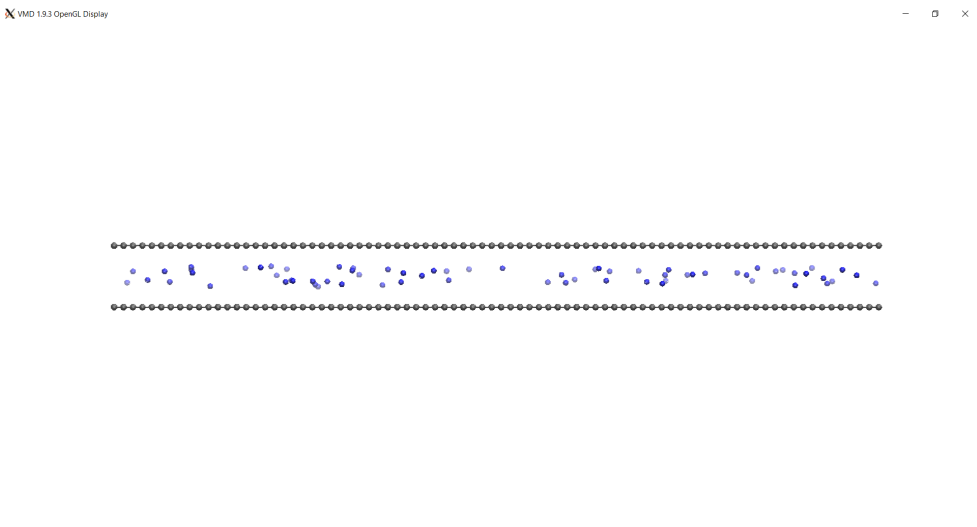

Figure S16. Molecular snapshot for H_2 adsorption on the graphene slit-shaped pore with an interlayer distance of 8 Å at a temperature of 300 K and a pressure of 70 atm. For clarity, the center of mass of the hydrogen molecules is pictured only (blue).

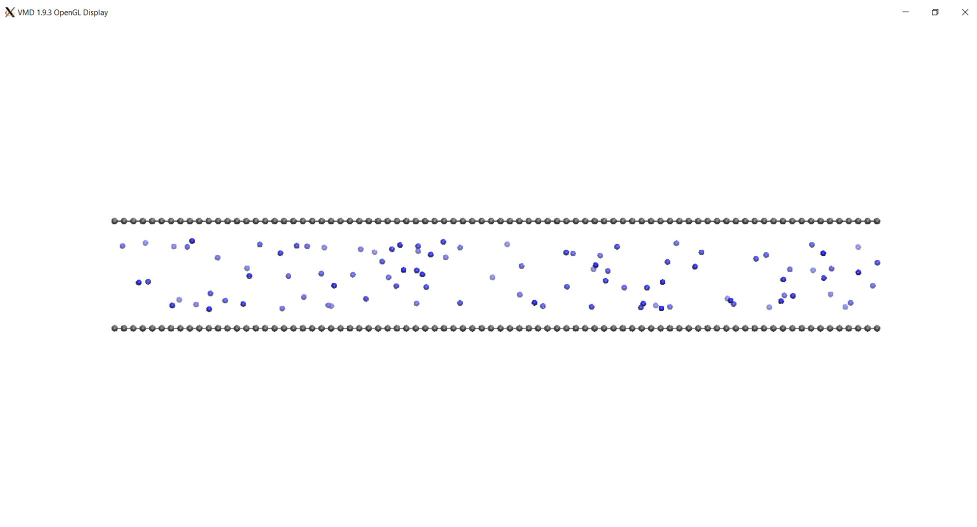

Figure S17. Molecular snapshot for H_2 adsorption on the graphene slit-shaped pore with an interlayer distance of 14 Å at a temperature of 300 K and a pressure of 70 atm. For clarity, the center of mass of the hydrogen molecules is pictured only (blue).

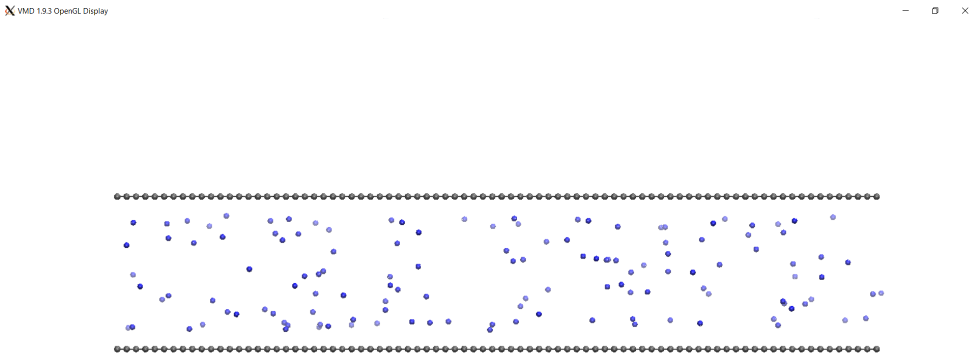

Figure S18. Molecular snapshot for H_2 adsorption on the graphene slit-shaped pore with an interlayer distance of 20 Å at a temperature of 300 K and a pressure of 70 atm. For clarity, the center of mass of the hydrogen molecules is pictured only (blue).

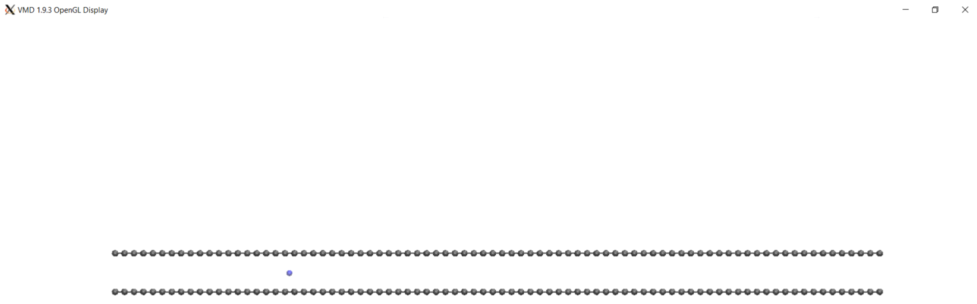

Figure S19. Molecular snapshot for CH_4/H_2 adsorption on the graphene slit-shaped pore with an interlayer distance of 5 Å at a temperature of 300 K and a pressure of 1 atm. For clarity, the center of mass of the hydrogen (blue) and methane (red) molecules is pictured only.

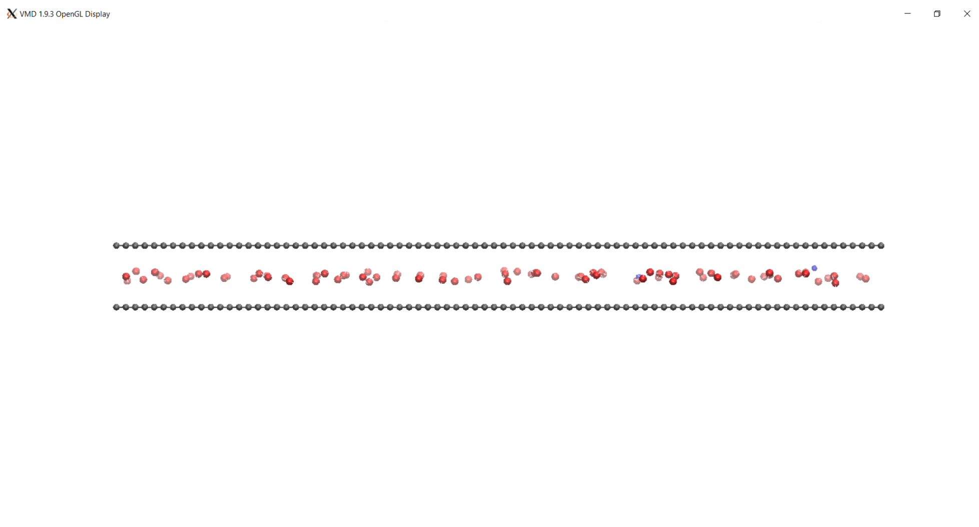

Figure S20. Molecular snapshot for CH_4/H_2 adsorption on the graphene slit-shaped pore with an interlayer distance of 8 Å at a temperature of 300 K and a pressure of 1 atm. For clarity, the center of mass of the hydrogen (blue) and methane (red) molecules is pictured only.

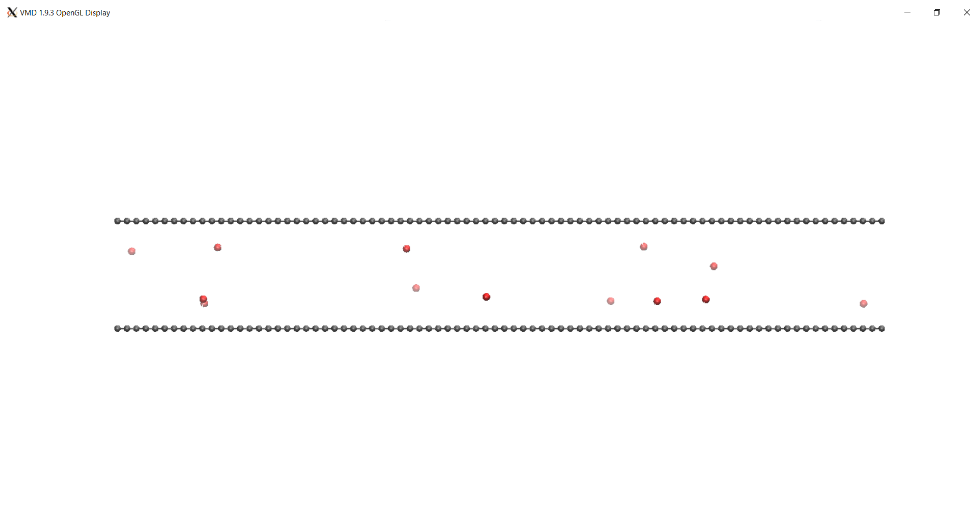

Figure S21. Molecular snapshot for CH_4/H_2 adsorption on the graphene slit-shaped pore with an interlayer distance of 14 Å at a temperature of 300 K and a pressure of 1 atm. For clarity, the center of mass of the hydrogen (blue) and methane (red) molecules is pictured only.

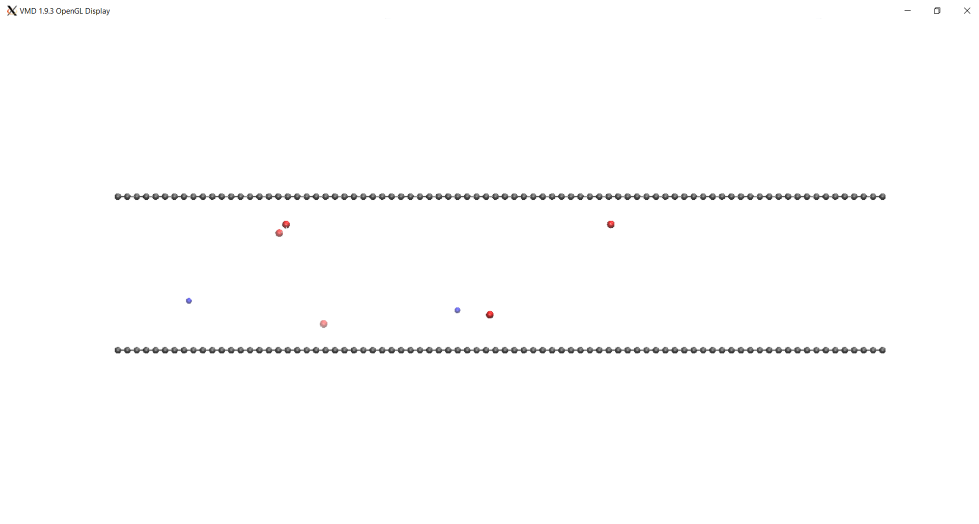

Figure S22. Molecular snapshot for CH_4/H_2 adsorption on the graphene slit-shaped pore with an interlayer distance of 20 Å at a temperature of 300 K and a pressure of 1 atm. For clarity, the center of mass of the hydrogen (blue) and methane (red) molecules is pictured only.

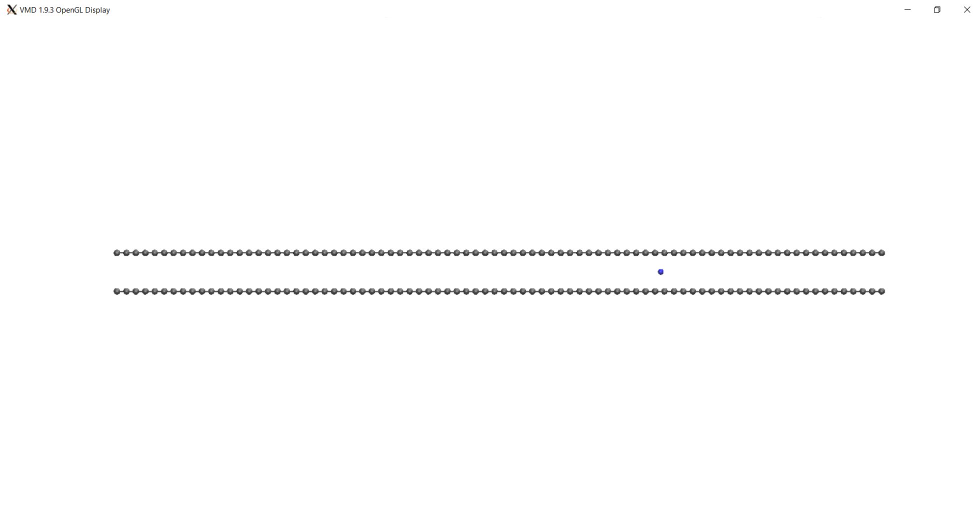

Figure S23. Molecular snapshot for CH_4/H_2 adsorption on the graphene slit-shaped pore with an interlayer distance of 5 Å at a temperature of 300 K and a pressure of 70 atm. For clarity, the center of mass of the hydrogen (blue) and methane (red) molecules is pictured only.

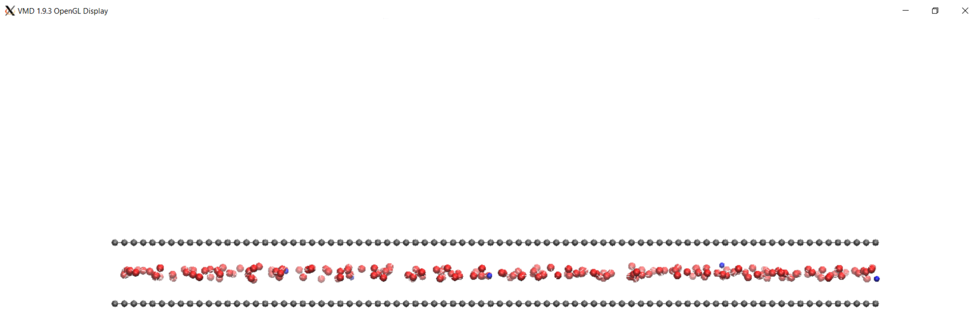

Figure S24. Molecular snapshot for CH_4/H_2 adsorption on the graphene slit-shaped pore with an interlayer distance of 8 Å at a temperature of 300 K and a pressure of 70 atm. For clarity, the center of mass of the hydrogen (blue) and methane (red) molecules is pictured only.

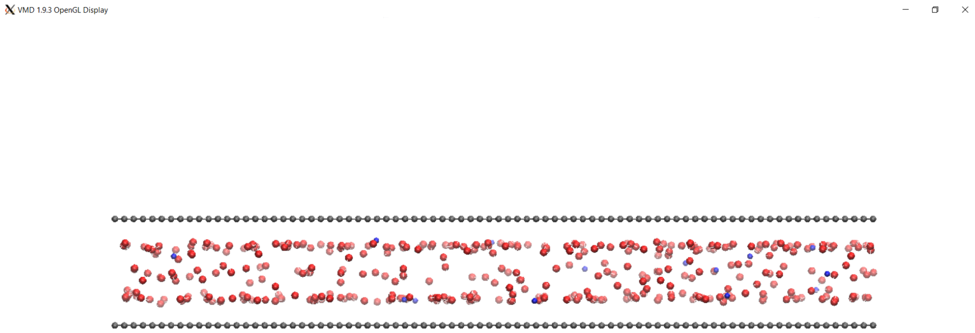

Figure S25. Molecular snapshot for CH_4/H_2 adsorption on the graphene slit-shaped pore with an interlayer distance of 14 Å at a temperature of 300 K and a pressure of 70 atm. For clarity, the center of mass of the hydrogen (blue) and methane (red) molecules is pictured only.

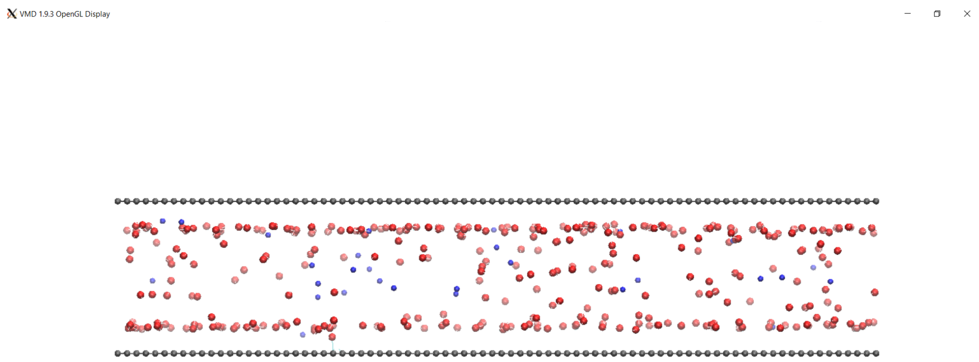

Figure S26. Molecular snapshot for CH_4/H_2 adsorption on the graphene slit-shaped pore with an interlayer distance of 20 Å at a temperature of 300 K and a pressure of 70 atm. For clarity, the center of mass of the hydrogen (blue) and methane (red) molecules is pictured only.
